# Hemorrhagic stroke treated by transcranial neuroendoscopic approach

**DOI:** 10.1038/s41598-021-90927-8

**Published:** 2021-06-04

**Authors:** Qiang Cai, Zhiyang Li, Wenju Wang, Baowei Ji, Junhui Liu, Zhibiao Chen, Qianxue Chen, Shanping Mao

**Affiliations:** 1 Department of Neurosurgery, Renmin Hospital of Wuhan University, No. 238, Jiefang Road, Wuchang District, Wuhan, 430060 Hubei China; 2 Department of Neurology, Renmin Hospital of Wuhan University, Wuhan, Hubei China

**Keywords:** Cerebrovascular disorders, Stroke

## Abstract

Hemorrhagic stroke (HS) is usually treated under microscopy, but recently, an increasing number of cases have been treated under neuroendoscopy. The objective of this study was to explore the feasibility and efficacy of a transcranial neuroendoscopic approach for HS. Based on etiology and clinical features, 203 HS patients were classified into two groups, with 100 patients in the primary HS (PHS) group and 103 patients in the secondary HS (SHS) group. All patients were treated either by full neuroendoscopy (FNE) or by neuroendoscopy combined with microsurgery (ECM). Outcomes were assessed according to the Glasgow Coma Scale (GCS) at discharge, and the rate of good plus excellent results was recorded as the GE rate to assess the treatment effect. All 203 patients underwent surgery successfully, with 165 patients who underwent FNE and 38 patients who underwent ECM. No patients died within 3 days after surgery, and the surgery-related mortality rate was 0%, but a total of 4 patients died by discharge, and the overall mortality rate was 1.97%. A total of 133 patients showed an excellent result and 16 showed a good result, for a total GE rate of 73%. Neuroendoscopy can provide excellent illumination, clear visualization, and multiangle views in HS. The transcranial neuroendoscopic approach is feasible and safe for both PHS and SHS and is very effective for hematoma evacuation. However, some aneurysms and most arteriovenous malformations and arteriovenous fistulas require ECM.

## Introduction

Hemorrhagic stroke (HS) is a devastating disease associated with high rates of mortality and disability; HS includes intracerebral hemorrhage (ICH) and subarachnoid hemorrhage (SAH)^1–5^. According to the latest data, stroke has risen to the second largest cause of death globally (5.5 million deaths), and this number could continue to rise with population aging. There were 80.1 million prevalent cases of stroke globally and 13.7 million new stroke cases in 2016^1^. The highest age-standardized incidence of stroke was observed in China (354 per 100,000 person-years). Although ischemic stroke accounts for 84.4% of cases of stroke, HS continues to be associated with the highest mortality rate among all forms of stroke and a substantial morbidity rate. In 2016, the number of global deaths due to ischemic stroke (2.7 million) was slightly lower than the number due to HS (2.8 million)^1^. Furthermore, ICH has a mortality rate of almost 50% when associated with intraventricular hemorrhage within the first month and an 80% rate of dependency at 6 months from onset^5^.

**Figure 1:**
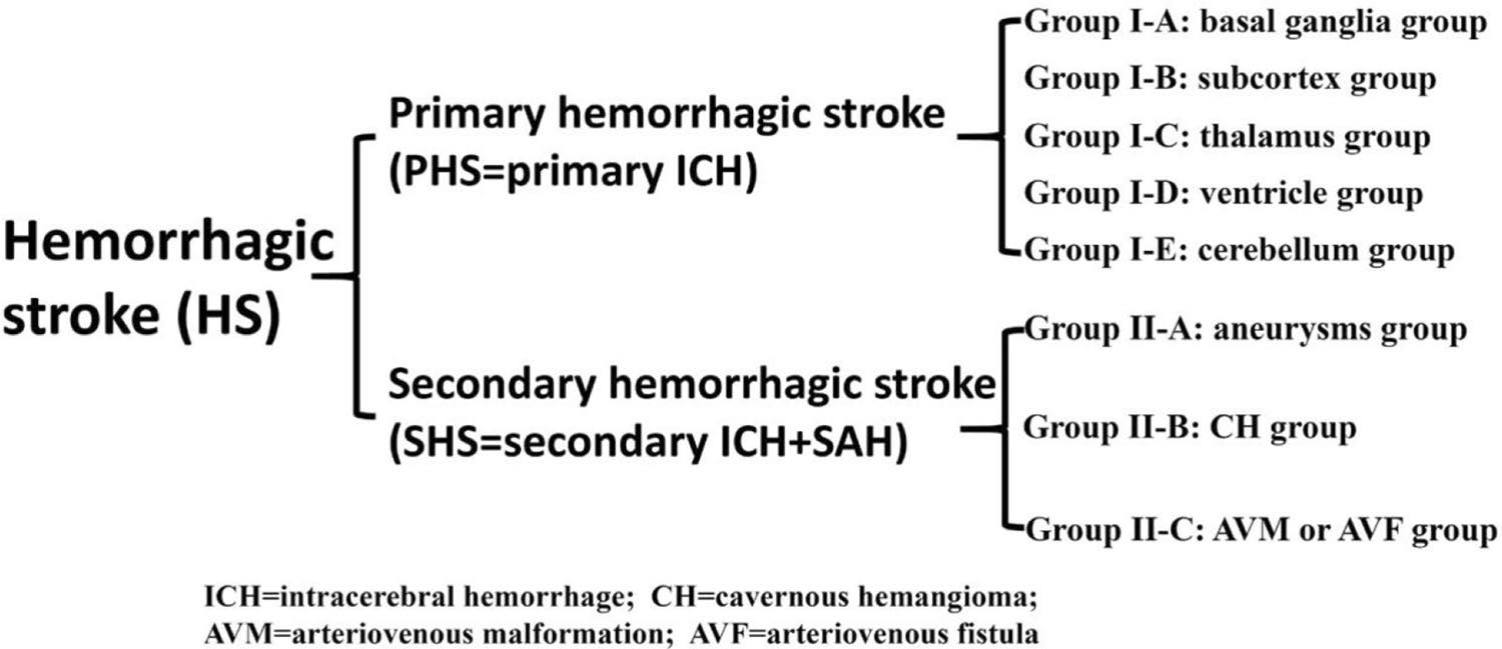
Classification chart of HS treated by a transcranial neuroendoscopic approach.

ICH can be classified into primary and secondary ICH based on the etiology, while SAH is a secondary disease mainly caused by aneurysms. Therefore, we classified HS patients into the primary HS (PHS) and secondary HS (SHS) groups, with the former comprising primary ICH cases and the latter comprising secondary ICH and SAH cases (Fig. 1).

Over the years, although considerable improvements have been achieved in the surgical treatment of HS, the strategy for optimal management remains controversial. To date, most cases of HS have been treated under microscopy. However, there has been a gradual increase in the number of cases treated by neuroendoscopy due to its advantages of excellent illumination, clear visualization, and multiangle views^6–10^. In this report, we present our preliminary experience with a transcranial neuroendoscopic approach in the treatment of HS. Satisfactory surgical and other outcomes were achieved in all cases in this study.

## Methods

### Surgical indications and patient selection

Between June 2016 and January 2020, 203 patients (107 males, 96 females; mean age: 57.3 years [range: 13–81 years]) with HS were surgically treated through a transcranial neuroendoscopic approach either by full neuroendoscopy (FNE) or by neuroendoscopy combined with microsurgery (ECM). These patients were classified into the PHS group (group I, 100 patients) and the SHS group (group II, 103 patients) according to the etiology. The PHS group was also classified into five subgroups according to the main location of the hematoma on computed tomography (CT): the basal ganglia group (group I-A, 59 patients); the subcortex group (group I-B, 19 patients); the thalamus group (group I-C, 12 patients); the ventricle group (group I-D, 5 patients); and the cerebellum group (group I-E, 5 patients) (Table 1). All patients underwent CT before and within 24 h after the operation, and the hematoma volume and evacuation rate were calculated by 3D Slicer software and the formula ([preoperative hematoma volume − postoperative hematoma volume]/preoperative hematoma volume) × 100%. The SHS group was classified into three subgroups according to the etiology: the aneurysm group (group II-A, 79 patients); the cavernous hemangioma (CH) group (group II-B, 13 patients); and the arteriovenous malformation (AVM) or arteriovenous fistula (AVF) group (group II-C, 11 patients) (Fig. 1).

**Table 1:** Demographics and HER of the 100 patients with PHS.

Groups (main location of hematoma)	Cases	Age	M:F	L:R	Pre-Vol	Post-Vol	HER
A (basal ganglia)	59	55.7	42:17	32:27	55.0	5.7	89.6
B (subcortex)	19	61.1	9:10	9:10	42.7	2.3	94.6
C (thalamus)	12	62.8	8:4	7:5	42.0	5.5	86.9
D (ventricle)	5	50.5	3:2	1:4	29.3	5.4	81.6
E (cerebellum)	5	70.2	2:3	1:4	18.8	0.9	95.2
Total	100	58.1	64:36	50:50	48.0	4.8	90.0

The cases in group D (ventricle) did not include hemorrhages originating from the thalamus, basal ganglia, subcortex or cerebellum that broke into the ventricles.

*GCS* Glasgow Coma Scale, *M:F* male:female, *L:R* left side:right side, *Pre* preoperation, *Post* postoperation, *Pre-Vol* preoperation volume (cm^3^), *Post-Vol* postoperation volume (cm^3^), *HER* hematoma evacuation rate (%).

The clinical data were collected, and the outcomes (including the hematoma evacuation rate [HER], perioperative mortality rate, and Glasgow Coma Scale [GCS] score at discharge) and complications were analyzed. The GCS score at discharge was recorded as 0 if the patient died at the hospital, and the outcome was recorded as “given up” when the GCS score was 3–5; “poor”, 6–8; “good”, 9–12; and “excellent”, 13–15. The rate of good plus excellent results in a group was considered the GE rate, which was used to analyze and evaluate the therapeutic efficacy of transcranial neuroendoscopy.

Patients with hemorrhage related to neoplasms, trauma, or bleeding disorders (i.e., a prothrombin time > 12.2 s, a partial thromboplastin time > 35.5 s, or a platelet count < 100 × 10^3^/mL) were excluded.

### Operative technique

**Figure 2:**
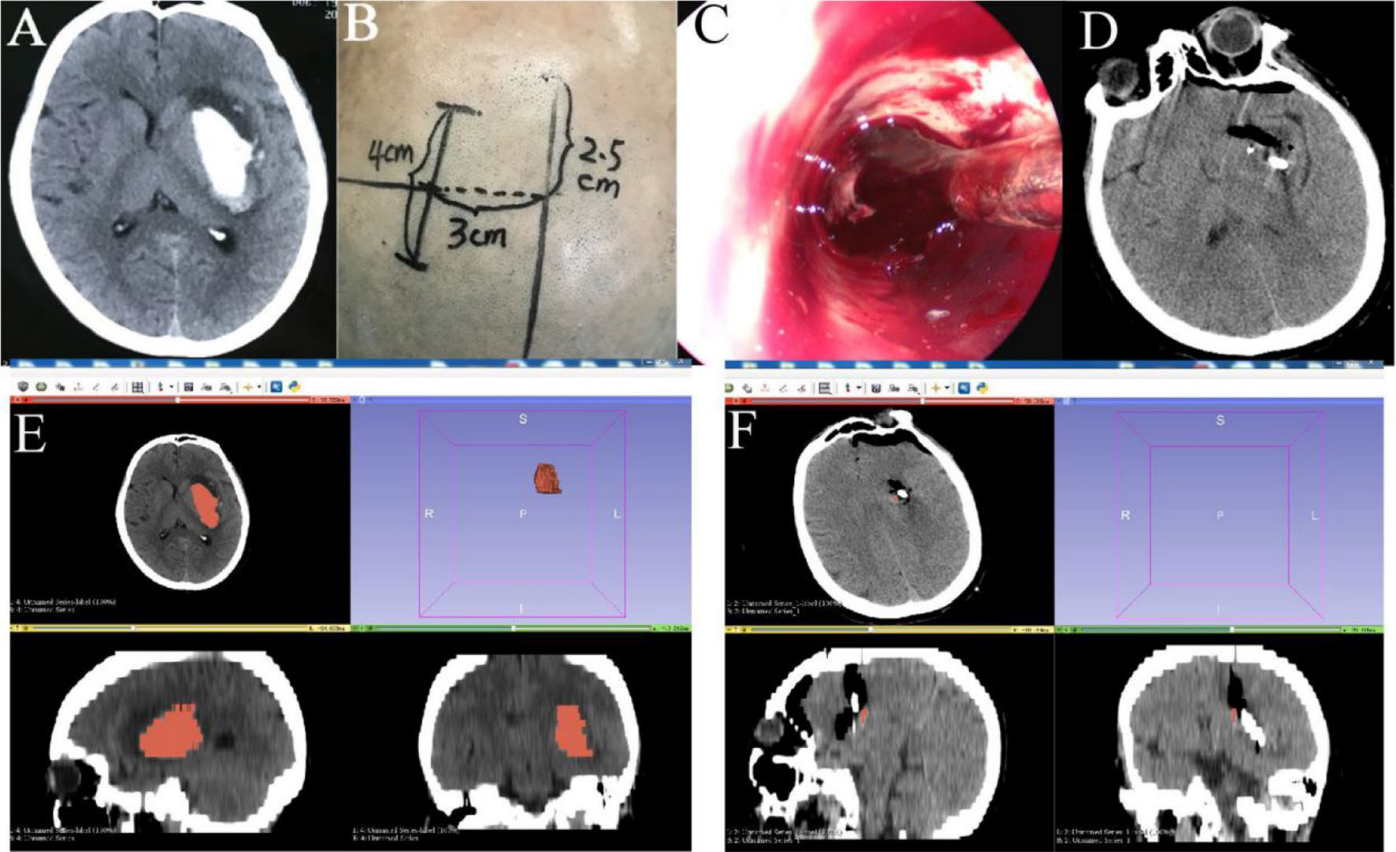
A left basal ganglia hemorrhage was evacuated by transcranial neuroendoscopy. (**A**) CT before the operation showed a hematoma located in the left basal ganglia. (**B**) A linear scalp incision of 4 cm was made. (**C**) The hematoma was evacuated under direct vision by neuroendoscopy. (**D**) Postoperative CT showed satisfactory evacuation of the hematoma. (**E**) The preoperative hematoma volume calculated by 3D Slice software was 23.5 ml. (**F**) The postoperative hematoma volume was 0.09 ml, and the evacuation rate was 99.6%.

**Figure 3:**
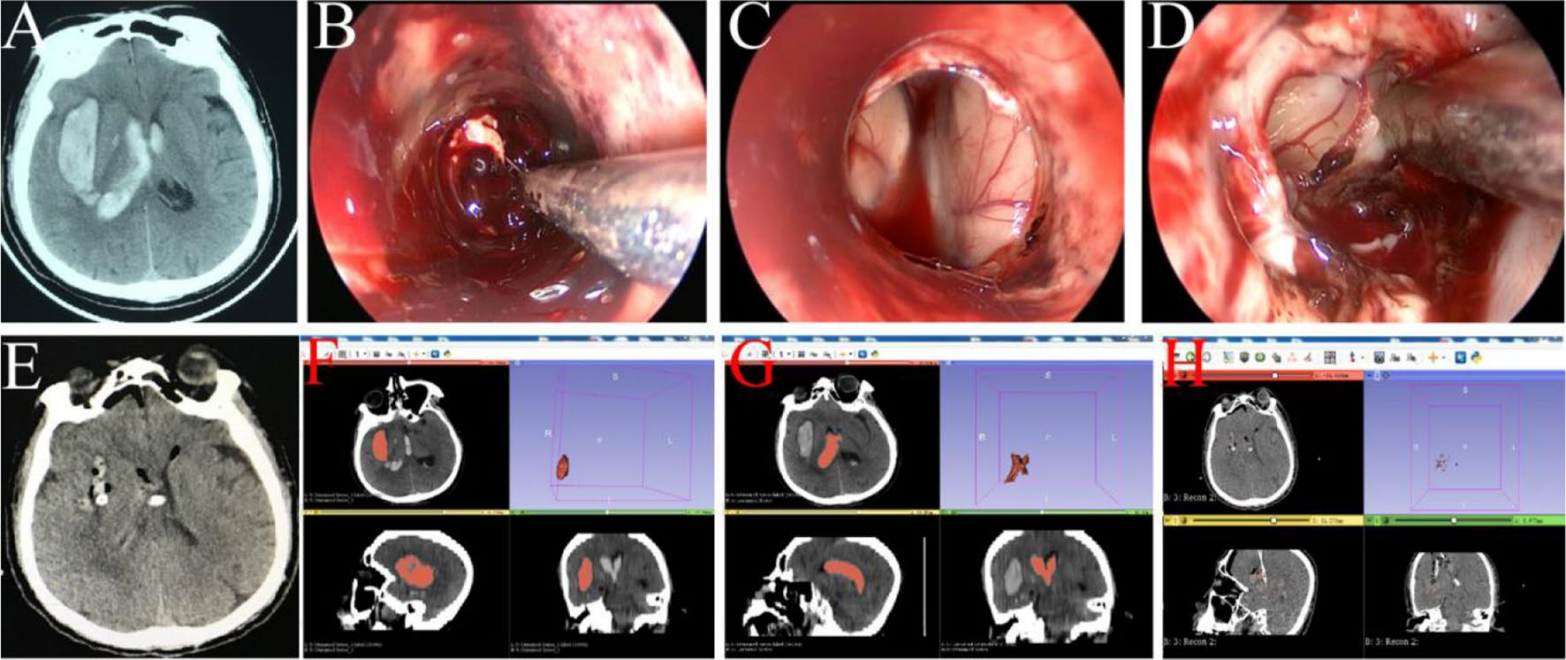
A right basal ganglia hemorrhage with extension into the ventricles was evacuated by transcranial neuroendoscopy. (**A**) CT before the operation showed a hematoma located in the right basal ganglia with extension into the ventricles. (**B**) The right basal ganglia hematoma was removed. (**C**) The hematoma in the right lateral ventricle was evacuated. (**D**) The hematoma in the left lateral ventricle was evacuated under neuroendoscopy through a transparent septum. (**E**) Postoperative CT showed satisfactory hematoma evacuation. (**F**) The preoperative volume of the hematoma in the right basal ganglia was 20.4 ml. (**G**) The preoperative volume of the hematoma in the ventricles was 21.7 ml. Thus, the total hematoma volume was 42.1 ml before the operation. (**H**) The postoperative hematoma volume was 1.41 ml, and the evacuation rate was 96.7%.

**Figure 4:**
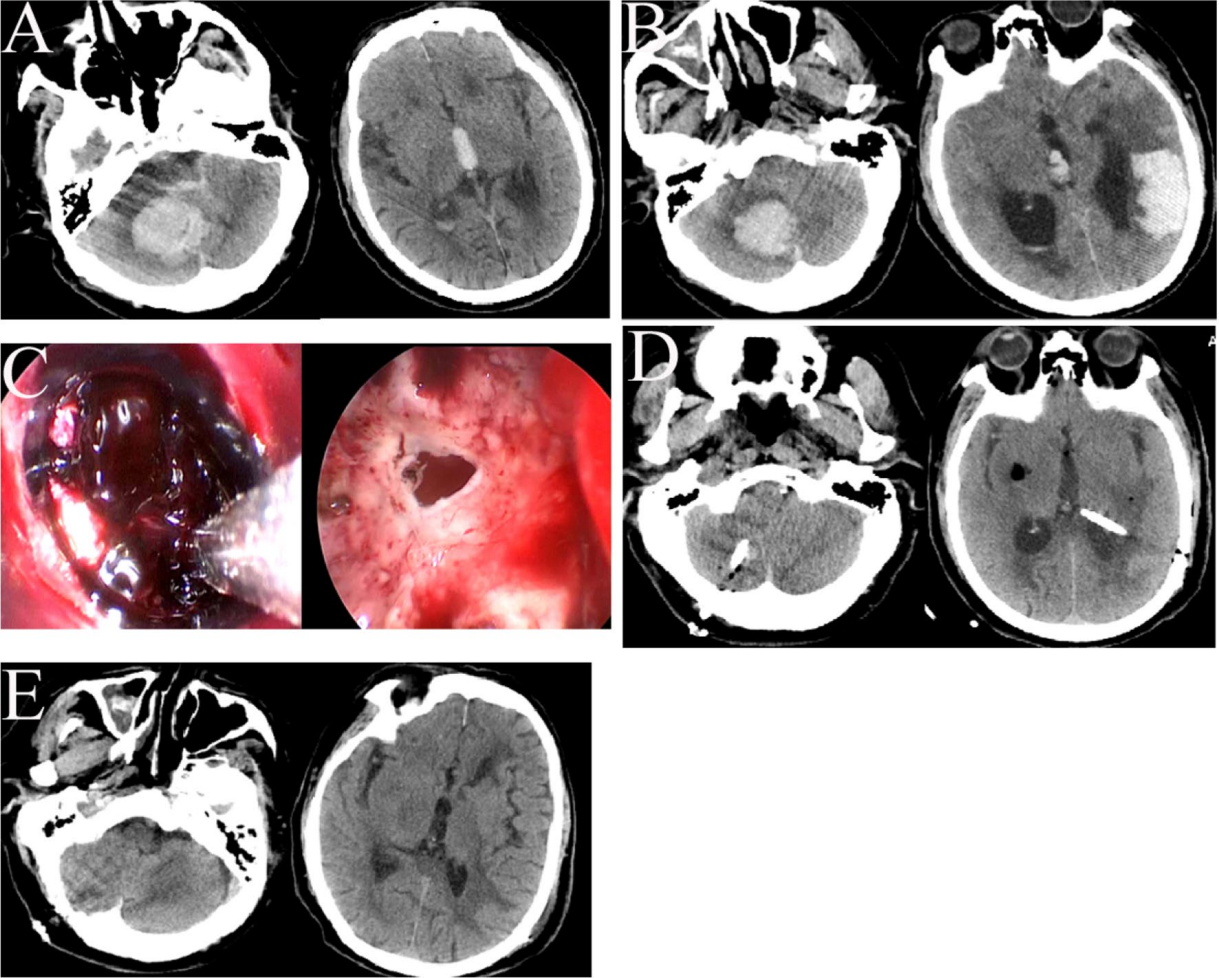
A right cerebellar hemorrhage and a left temporal lobe hematoma were evacuated by transcranial neuroendoscopy. (**A**) CT showed a hematoma in the right cerebellum at admission. (**B**) Two days later, a temporal lobe hematoma began to emerge, as well as hydrocephalus. (**C**) The left temporal lobe and right cerebellum hematomas were removed under neuroendoscopy. (**D**) CT showed excellent hematoma removal on the day after the operation. (**E**) CT showed no signs of hematoma before discharge.

For PHS, the surgical approach was selected according to the location of the hematoma. Usually, a linear scalp incision of approximately 4–5 cm and a cortical incision of 1.5–2 cm were made, and then a transparent plastic sheath was inserted into the hematoma cavity. Next, a 0° rigid neuroendoscope (Karl Storz, Germany) was introduced into the hematoma cavity, and the hematoma was removed under direct vision by neuroendoscopy. The bleeding site was coagulated by a bipolar coagulator, and finally, a soft catheter was placed into the hematoma cavity to drain any residual liquid (Figs. 2A–F, 3A–H, 4A–E).

**Figure 5:**
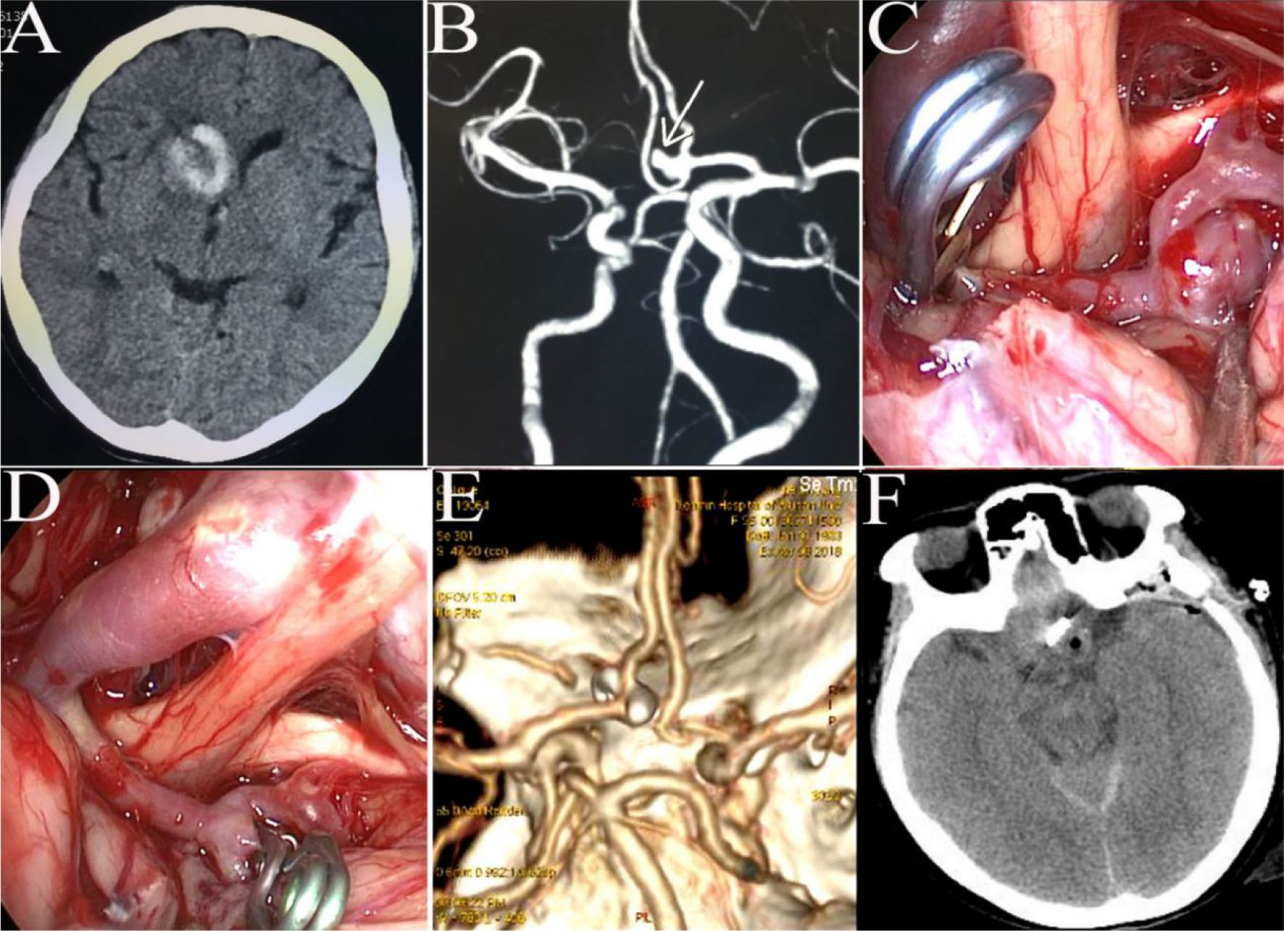
An ACoA aneurysm was clipped under neuroendoscopy. (**A**) Preoperative CT showed a small hematoma in the basal ganglia and right lateral ventricle. (**B**) Preoperative CT angiography (CTA) showed a saccular aneurysm at the ACoA complex. (**C**) Left A1 was clipped temporarily, and the aneurysm, as well as the left and right A2, were exposed. (**D**) The aneurysm was clipped under neuroendoscopy, and the temporary clip was removed. (**E**) Postoperative CTA showed complete clipping of the aneurysm. (**F**) Postoperative CT showed proper placement of the aneurysm clip.

**Figure 6:**
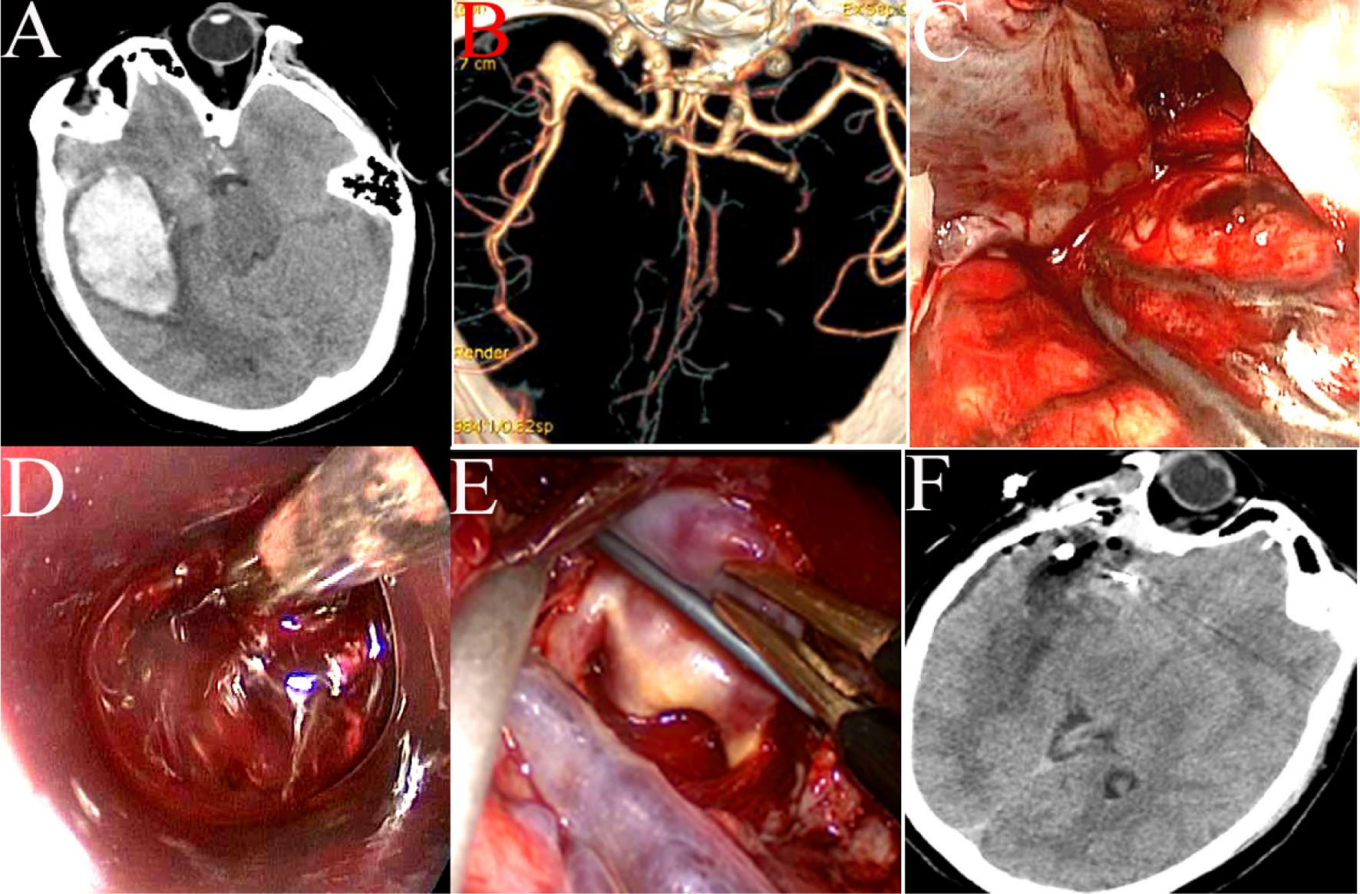
A ruptured MCA aneurysm with a hematoma was clipped under neuroendoscopy. (**A**) Preoperative CT showed a large hematoma in the right temporal lobe. (**B**) Preoperative CTA showed a saccular aneurysm at the right MCA bifurcation. (**C**) The brain pressure was high after opening the dura. (**D**) The hematoma was evacuated under neuroendoscopy. (**E**) The aneurysm was exposed and clipped under neuroendoscopy. (**F**) Postoperative CT showed complete removal of the hematoma and proper placement of the clip.

A pterional approach was employed for anterior circulation aneurysms, and a far-lateral surgical approach was used for aneurysms of the posterior inferior cerebellar artery (PICA). After opening the dura mater, a 30° or 0° rigid neuroendoscope was introduced into the operating field. The arachnoid membrane was then sharply dissected to expose the aneurysm(s). After the neck was separated carefully, the aneurysm was completely clipped, and the parent artery and perforating branches were carefully checked under neuroendoscopy. For aneurysms with a hematoma, the hematoma was first removed under neuroendoscopy to reduce intracerebral pressure (Figs. 5A–F, 6A–F). During surgery, a microscope was prepared in case of intraoperative aneurysm rupture with excessive bleeding. In such cases, we immediately switched to microscopic surgery.

**Figure 7:**
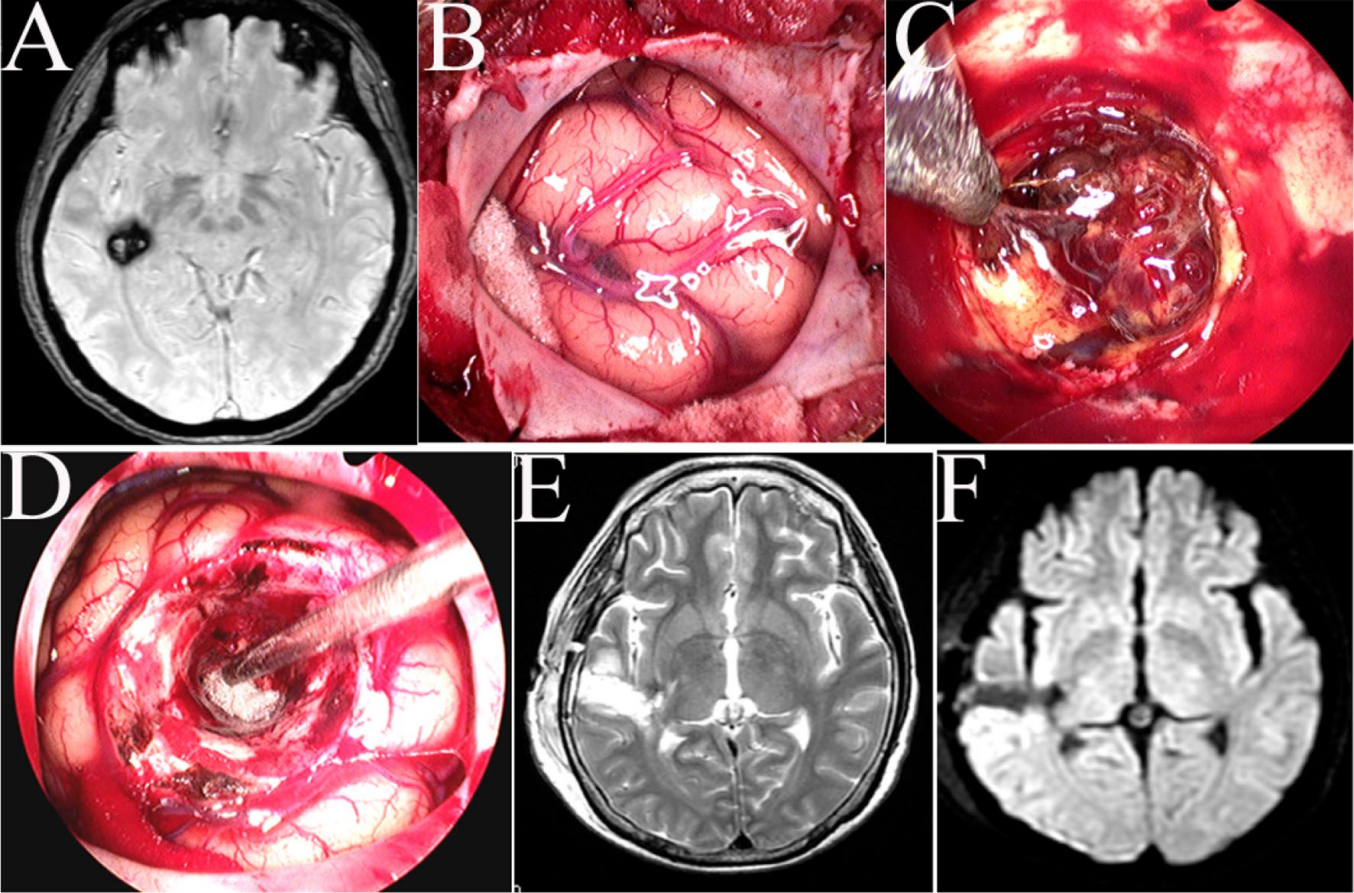
A patient with a small cavernous hemangioma in the right medial temporal lobe was treated under neuroendoscopy. (**A)** Preoperative magnetic resonance imaging (MRI) showed a small cavernous hemangioma in the right temporal lobe. (**B**) Cross incision of the dura matter to expose the temporal lobe. (**C**) The cavernous hemangioma was removed under direct visualization by neuroendoscopy. (**D**) The cavity of the cavernous hemangioma was checked. (**E**,**F**) T2-weighted axial and SWAN postoperative MRI showed complete removal of the cavernous hemangioma.

For CH, a transcranial neuroendoscopic approach combined with a trans-endoport technique was used. First, a 4- to 5-cm craniotomy was created, followed by a 1- to 2-cm linear corticectomy. The film of the endoport was rolled into a thin stick, used to penetrate the brain and then dilated to 2 cm. The lesions were coagulated, dissected, and removed under direct visualization by neuroendoscopy (Fig. 7A–F).

**Figure 8:**
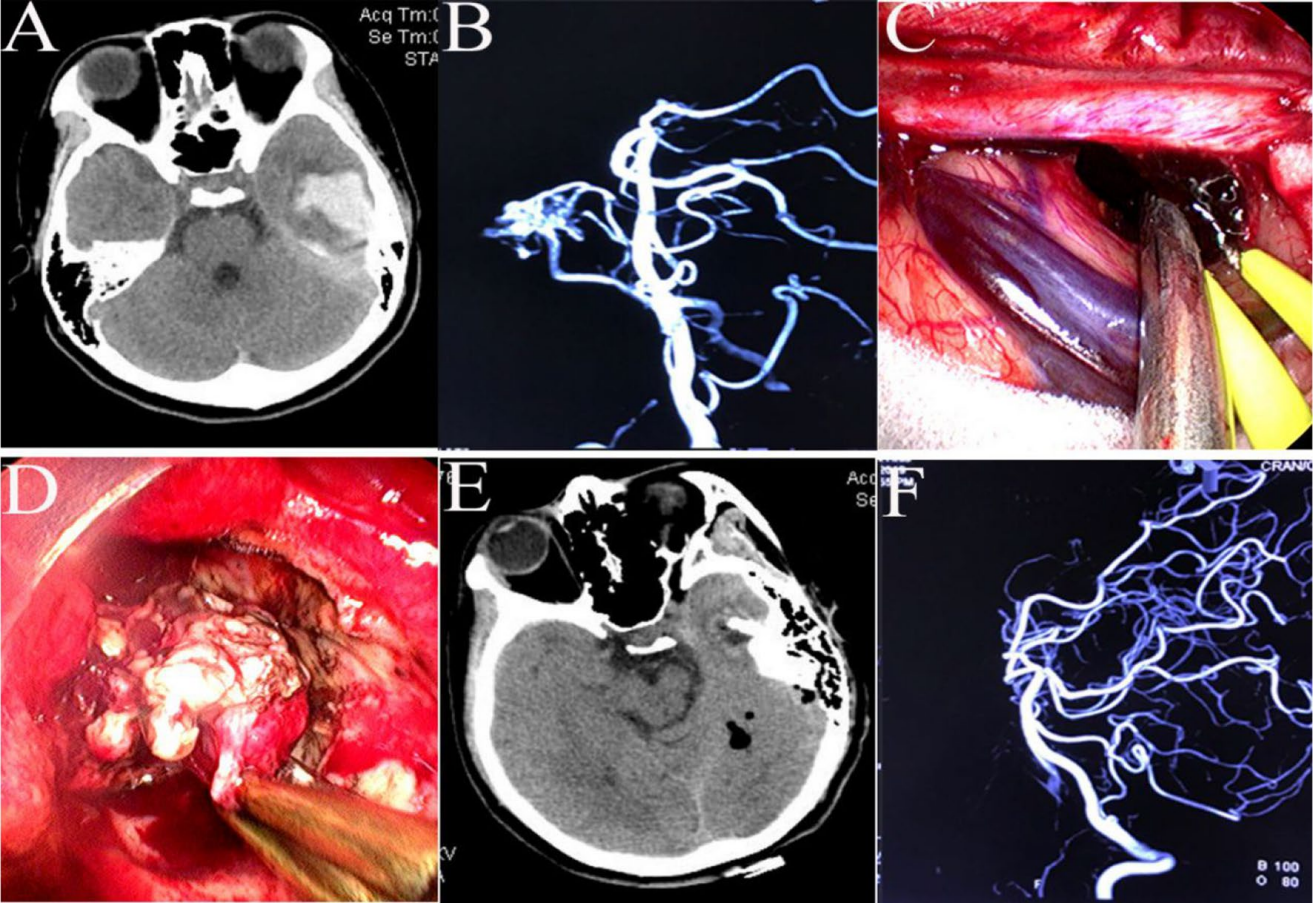
An AVM and hematoma were removed under neuroendoscopy. (**A**) CT showed a small hematoma at the back of the temporal lobe. (**B**) Digital subtraction angiography (DSA) showed a small AVM in the temporal lobe. (**C**) The hematoma was evacuated under neuroendoscopy. (**D**) The nidus of the AVM was circumferentially dissected under neuroendoscopy. (**E**) Postoperative CT showed complete removal of the hematoma. (**F**) Postoperative DSA showed no signs of the AVM.

For small AVMs with a hematoma, a neuroendoscope was employed to first evacuate the hematoma; then, the AVM core, feeding artery, and draining vein were identified, cauterized, isolated, divided, and removed (Fig. 8A–F). These techniques were also used for hematomas caused by AVFs. However, for larger AVMs, neuroendoscopy was only employed to remove the hematoma, and the AVM was then removed under microscopy.

For PHS, the surgeon held the scope most of the time, but the assistant held the scope when the operation became difficult so a bimanual technique could be applied by the surgeon. For SHS, an assistant was employed to hold the scope, and the surgeon operated with both hands. This study was approved by the ethics committee of Renmin Hospital of Wuhan University (Wuhan, China). Written informed consent for publication was obtained from the patients or their family members. All procedures performed on human participants were in accordance with the ethical standards of the institutional and/or national research committees and the 1964 Helsinki Declaration and its later amendments or comparable ethical standards. Written informed consent was obtained from the patients or their family members.

### Ethical approval

All procedures performed in studies involving human participants were in accordance with the ethical standards of the institutional and/or national research committee and with the 1964 Helsinki declaration and its later amendments or comparable ethical standards.

### Informed consent

Informed consent was obtained from all individual participants included in the study.

## Results

A total of 203 patients were selected and analyzed, including 100 PHS patients (49.3%) and 103 SHS patients (50.7%). All PHS patients underwent surgery by FNE, and the average HER was 90%. The highest evacuation rate was 95.2% in the cerebellum group, followed by 94.6% in the subcortex group, 89.6% in the basal ganglia group, 86.9% in the thalamus group, and 81.6% in the ventricle group (Table 1). Although the ventricle group had the lowest evacuation rate, it had the highest GCS score improvement of 5.0. The lowest GCS score improvement was only 0.7 in the thalamus group, which could be attributed to the important function of the thalamus. The GE rate for PHS was 90% in the subcortex group, 80% in the ventricle group, 80% in the cerebellum group, 61% in the basal ganglia group, and 43% in the thalamus group. Thus, transcranial neuroendoscopy was a superior technique for treating subcortical, intraventricular, and cerebellar hemorrhage, a good technique for treating basal ganglia hemorrhage, and a poor technique for treating thalamic hemorrhage (Tables 1, 2).

**Table 2:** Outcome at discharge and GCS score improvement of the 100 patients with PHS.

Groups (main location of hematoma)	Cases	Pre-GCS	Post-GCS	GCS Imp	Outcome at discharge					
					Dead	Give up	Poor	Good	Excellent	GE rate (%)
A (basal ganglia)	59	7.6	10.6	3.0	1	12	10	6	30	61
B (subcortex)	19	9.1	13.4	4.3	0	1	1	3	14	90
C (thalamus)	12	6.7	7.4	0.7	2	2	2	2	3	43
D (ventricle)	5	7.6	12.6	5.0	0	0	1	1	3	80
E (cerebellum)	5	10.8	12.8	2.0	0	0	1	0	4	80
Total	100	7.9	10.9	3.0	3	15	15	12	54	66

*GCS* Glasgow Coma Scale, *Pre* preoperation, *Post* postoperation, *Pre-GCS* preoperation GCS, *Post-GCS* postoperation, *GCS Imp* GCS improvement, *GE rate* percentage of good and excellent results in that group.

In the SHS group, there were a total of 88 aneurysms among the 79 patients, which included 27 posterior communicating artery aneurysms, 26 middle cerebral artery aneurysms, 20 anterior communicating artery aneurysms, 5 A1-A4 aneurysms, 3 internal carotid artery bifurcation aneurysms, 2 anterior choroidal artery aneurysms, 1 ophthalmic artery aneurysm, and 2 PICA aneurysms. The Hunt and Hess (H–H) grade was assigned as follows: I in 14 cases, II in 45 cases, III in 11 cases, IV in 8 cases, and V in 1 case (Table 3). All aneurysm patients underwent successful surgical treatment, with 49 patients treated by FNE and 30 treated by ECM. None of these patients showed cerebrospinal fluid (CSF) leakage, and none died. The GE rate was also closely related to the H–H grade, which was 93% for grade I, 96% for grade II, 64% for grade III, 13% for grade IV, and 0% for grade V, with an average of 81% for all aneurysm cases (Table 3).

**Table 3:** Hunt-Hess grade, clinical characteristics and outcome of the 79 patients with aneurysms.

H–H grade	Cases	Age	M:F	FNE	Hema	Outcome at discharge					
						Dead	Give up	Poor	Good	Excellent	GE rate (%)
I	14	58.1	6:8	13	0	0	0	1	0	13	93
II	45	59.0	13:32	28	1	0	2	0	1	42	96
III	11	56.3	7:4	5	4	0	1	3	1	6	64
IV	8	60.6	2:6	3	7	0	6	1	0	1	13
V	1	61.0	1:0	0	1	0	0	1	0	0	0
Total	79	58.7	29:50	49	13	0	9	6	2	62	81

*H–H grade* Hunt-Hess grade, *M:F* male:female, *FNE* full neuroendoscopy, *Hema* accompanying hematoma.

Twelve of the 13 CH cases were treated by FNE, and one was treated by ECM. None of the patients died, and the GE rate was 92% (Table 4). In the AVM/AVF group, there were 9 cases of AVM and 2 cases of AVF, and all patients presented with accompanying hematomas. All AVM/AVF patients were treated successfully, 4 by FNE and 7 by ECM. One patient died because of postoperative complications of multisystem organ failure, and the GE rate was only 64% in this group (Table 4).

**Table 4:** Demographics, clinical characteristics and outcome of the 103 patients with SHS.

Groups (according to etiology)	Cases	Age	M:F	FNE	Hema	Outcome at discharge					
						Dead	Give up	Poor	Good	Excellent	GE rate (%)
A (aneurysms)	79	58.7	29:50	49	13	0	9	6	2	62	81
B (CH)	13	48.9	6:7	12	3	0	0	1	1	11	92
C (AVM/AVF)	11	49.5	8:3	4	11	1	1	2	1	6	64
Total	103	56.5	43:60	65	27	1	10	9	4	79	81

*AVM* arteriovenous malformation, *AVF* arteriovenous fistula, *CH* cavernous hemangiomam, *M:F* male:female, *FNE* full neuroendoscopy, *Hema* accompanying hematoma.

Overall, all 203 patients underwent successful surgery, 165 patients by FNE and 38 by ECM. No patients died within 3 days after surgery, and the surgery-related mortality was 0%, but a total of 4 patients died by discharge, for an overall mortality rate of 1.97%. This mortality rate refers to patients who died in the hospital and does not include patients who died at home or in the community after discharge. A total of 133 patients showed an excellent result, and 16 showed a good result, for a total GE rate of 73% (Table 5).

**Table 5:** Demographics, clinical characteristics and outcome of the 203 patients with HS.

Groups (according to etiology)	Cases	Age	M:F	FNE	Hema	Outcome at discharge					
						Dead	Give up	Poor	Good	Excellent	GE rate (%)
I (PHS)	100	58.1	64:36	100	100	3	15	15	12	54	66
II (SHS)	103	56.5	43:60	65	27	1	10	9	4	79	81
Total	203	57.3	107:96	165	127	4	20	24	16	133	73

*AVM* arteriovenous malformation, *AVF* arteriovenous fistula, *CH* cavernous hemangioma, *M:F* male:female, *FNE* full neuroendoscopy, *Hema* accompanying hematoma.

## Discussion

Stroke can be further categorized into ischemic stroke (IS), ICH, SAH, and stroke of undetermined type (UND), which account for 69.6%, 23.8%, 4.4%, and 2.1%, respectively, of stroke cases in China^3^. While ICH can also be classified into primary and secondary ICH, the former is caused mostly by hypertension or cerebral amyloid angiopathy, while the latter is usually caused by AVM, CH, aneurysms or tumors^11^.

Although HS includes ICH and SAH, the etiology and treatment strategy for secondary ICH are very different from those for primary ICH but similar to those for SAH. Therefore, we classified HS into two groups, PHS (primary ICH) and SHS (secondary ICH + SAH); these new groups are more consistent with their clinical and surgical features. This method also simplified the traditional classification of stroke^5,11,13^ from four types to three types, i.e., IS, HS (PHS + SHS), and UND, and facilitated our analysis and understanding of this complex disease.

To date, three main surgical methods have been applied for treating PHS, i.e., open craniotomy, stereotactic aspiration, and neuroendoscopic surgery, each with its advantages, shortcomings and indications^12^. Open craniotomy causes excessive damage, while stereotactic aspiration results in a high rebleeding rate^12–14^. Neuroendoscopic surgery is also a minimally invasive approach that has a higher evacuation rate, better functional outcomes and lower rates of complications and mortality due to its advantages of excellent illumination, multiangle views and close observation^15,16^. Nishihara et al.^17^ achieved almost complete hematoma evacuation (86–100%), and others have reported evacuation rates from 87.0 to 99.0%^6,7^. The rebleeding rate was decreased to 0% by Qiu et al.^18^, and the overall rebleeding rate was 0–3.3%, which is much lower than that of other approaches^12,13^. In our case series, we also found that the neuroendoscopic approach had a better evacuation rate, fewer complications, and a lower mortality rate.

The most common causes of SHS are aneurysms, AVM/AVF, and CH, among others^5^. Aneurysms are traditionally treated by clipping under microscopy or by endovascular techniques. Although endovascular techniques yield excellent results in most cases, the clipping technique remains the gold standard, especially for ruptured aneurysms combined with hematomas. Currently, almost all aneurysm clipping is performed under microscopy. However, the associated shortcomings include only a straight-line view, weak illumination in deep surgical fields, and the relatively low magnification of the microscope; thus, inadequately aneurysm clipping and parent vessel occlusion can occur^19,20^. To overcome these shortcomings, the neuroendoscope has gradually been introduced to aneurysm clipping. In 2006, Kassam et al.^21^ first reported the clipping of a coiled vertebral artery aneurysm under endoscopy by the endonasal endoscopic approach (EEA). Their report was followed by reports from several other surgeons, which demonstrated the feasibility of this technique. However, the inherent defect of the EEA is its relatively high rate of CSF leakage. Thus, an argument against the EEA in aneurysm clipping arose, leading to the application of transcranial approaches, as reported by a few surgeons. In Wong’s review, Perneczky performed seven aneurysm clippings exclusively using an endoscope through a pterional craniotomy, and Radovanovic clipped aneurysms by an endoscopic approach through a small craniotomy^22^. In our case series, we successfully operated on 79 patients with aneurysm clipping by a transcranial neuroendoscopic approach. No patients showed CSF leakage, which demonstrates that this approach is feasible and safe for aneurysms.

AVMs/AVFs remain challenges due to their annual bleeding rates and relatively high morbidity and mortality rates^23^. Microsurgery is the most common treatment because of its immediate curative effect and ability to simultaneously remove AVMs and hematomas. To date, all AVMs/AVFs have been resected under microscopy, and treatment under neuroendoscopy has not been attempted in any case. In this study, we treated ruptured AVMs/AVFs with hematomas by FNE (four cases) or ECM (seven cases). This shows that this approach is feasible for such conditions. However, for small AVMs/AVFs in our series, we believe that further evaluation is needed to prove the merit of this approach.

CH is an uncommon neurovascular condition that can lead to SHS, and surgical treatment is usually performed under microscopy. We treated 13 cases of CH by transcranial neuroendoscopy with the trans-endoport technique. All patients successfully underwent neuroendoscopic surgery, and no perioperative deaths occurred. These findings indicate that this approach might be a viable and effective method for treating CH.

## Conclusions

Neuroendoscopy can provide excellent illumination, clear visualization, and multiangle views in HS patients. The transcranial neuroendoscopic approach is feasible and safe for the treatment of both PHS and SHS and is very effective for hematoma evacuation. In PHS, transcranial neuroendoscopy was a superior technique in cases of subcortical, intraventricular, and cerebellar hemorrhage and a good technique in cases of basal ganglia hemorrhage. In SHS, transcranial neuroendoscopy was superior in cases of CH and aneurysms and good in cases of AVMs/AVFs. However, some aneurysms and most AVMs/AVFs require ECM.
